# Promotion of SO_2_ resistance of Ce–La/TiO_2_ denitrification catalysts by V doping

**DOI:** 10.1039/d3ra07073e

**Published:** 2024-01-10

**Authors:** Yang Liu, Na Wang, Huidong Xie, Yepeng Sun, Kaiyue Yang, Liang Zhang, Chang Yang, Chengmin Ge

**Affiliations:** a Xi'an University of Science and Technology Xi'an 710054 Shaanxi China; b Shaanxi University of Science and Technology Xi'an 710016 Shaanxi China wangna811221@sust.edu.cn +86-29-82202335 +86-29-82203378; c Xi'an University of Architecture and Technology Xi'an 710055 Shaanxi China; d Shandong Dongyuan New Material Technology Co. 257300 Shandong China

## Abstract

Conventional cerium-based denitrification catalysts show good catalytic activity at moderate and high temperatures, but their denitrification performance may be decreased due to poisoning by SO_2_ in the flue gas. In this paper, V was introduced into Ce–La/TiO_2_ catalysts by a ball-milling method, and the effects of the V content on catalyst denitrification performance and SO_2_ resistance were investigated. Fourier-transform diffuse reflectance *in situ* infrared spectroscopy was used to examine the denitrification mechanism and evaluate the catalysts for surface acidity, redox characteristics, and SO_2_ adsorption. After introducing V, Brønsted acids played the dominant role in the catalytic reaction by increasing the number of acidic sites on the catalyst surface, adsorbing NH_3_ to participate in the reaction, and improving the sulfur resistance by inhibiting SO_2_ poisoning. The Ce^3+^ and O ratio on the catalyst surface were also enhanced by V doping, which reduced interactions between SO_2_ and the primary metal oxide active ingredients. The modified catalyst inhibited the formation of sulfate species on the catalyst surface and prevented the generation of additional nitrate species on the surface, which protected the main active sites. After V doping, the NH_3_-SCR reaction on the catalyst surface followed the Langmuir–Hinshelwood mechanism.

## Abstract 2

Ce–La–V/TiO_2_ was prepared using cerium-based catalysts and the effect of the V active component tested. The mechanism of the catalyst's resistance to SO_2_ toxicity at high temperatures was elucidated using various physicochemical characterization techniques and reaction kinetics analysis.

## Graphical abstract

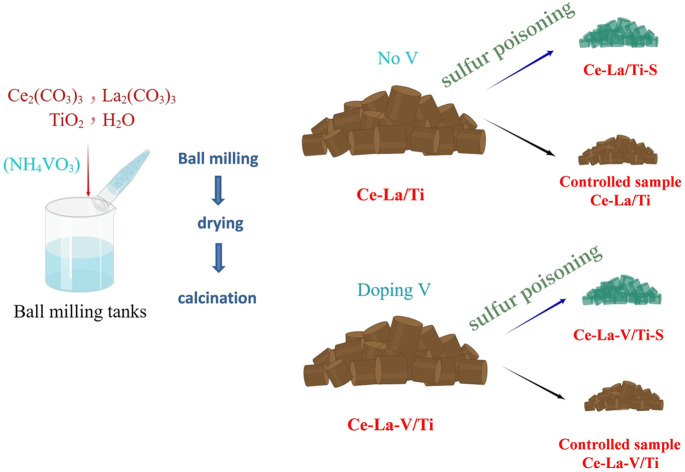

## 1 Introduction

NO_*x*_ is a major atmospheric pollutant whose emissions cause acid rain, photochemical smog, and ozone layer depletion.^1^ Selective catalytic reduction (SCR) has a wide range of denitrification efficiencies, does not produce secondary contamination due to NH_3_ escape, and is widely used in various plants and municipal waste facilities.^2^ Cerium-based catalysts are one of the commonly used catalysts in SCR, which is due to the fact that Ce atoms contain a special arrangement of electrons outside the nucleus and have excellent oxidizing properties and oxygen storage capacity. CeO_2_ can be converted from Ce^4+^ to Ce^3+^ when cerium ions generate oxygen vacancies,^3^ giving the catalyst excellent redox properties and thermal stability. Cerium promotes the conversion of nitrate species and ammonia species, which facilitates the conversion of NH_3_ and NO to reactive intermediates on the catalyst surface, thus promoting the NH_3_-SCR reaction.

Usually, the flue gas in coal-fired boilers contains SO_2_, which inevitably poisons the catalyst and reduces its denitrification performance. The reasons for SO_2_ poisoning can be classified into three categories: (1) the competitive adsorption of SO_2_ with reactants inhibits the formation of reactive intermediates; (2) the reaction of SO_2_ with NH_3_ to generate (NH_4_)_2_SO_4_ and NH_4_HSO_4_, which covers active sites on the catalyst surface and reduces the specific surface area of the catalyst; (3) metal active sites are sulfated by SO_2_ to create metal sulfates, which results in the loss of the active components and results in irreversible deactivation. For Ce-based catalysts, SO_2_ can be adsorbed and oxidized to SO_3_ by CeO_2_ to generate Ce(SO_4_)_2_ or Ce_2_(SO_4_)_3_ or react with NH_3_ to form stable (NH_4_)_2_SO_4_ or NH_4_HSO_4_, the NO conversion of CeO_2_/TiO_2_ catalysts was close to 100% above 300 °C and remained stable for 12 h. However, the value decreased significantly after 12 h, which was below 40% after 48 h.^4^ For Mn-based catalysts, the NO conversion of fresh samples of MnO_2_-based catalysts was 100% at 130–150 °C. After SO_2_ poisoning, the NO conversion decreased sharply from 100% to 53% at 130 °C and did not increase when the reaction temperature was increased to 150 °C.^5^ Ma *et al.*^6^ prepared Cu_0.02_Fe_0.2_W_0.02_TiO_*x*_ by doping W to Cu_0.02_Fe_0.2_TiO_*x*_ catalysts, the NO conversion of which maintained at 100% after passing water and sulfur at 240 °C for 10 h. Jiang *et al.*^7^ prepared Mn–Fe(0.4)–Ce(0.4)/ACN catalysts, which showed a NO_*x*_ conversions higher than 95% at 100–250 °C, and remained around 91% at 175 °C for 32 h under 100 ppm SO_2_ and 10 vol% H_2_O.

Early studies on SO_2_ poisoning catalysts mostly concentrated on vanadium-based catalysts. In the temperature range of 180 to 400 °C, Li *et al.*'s^8^ investigation of the distribution of SO_2_ oxidation products during the NH_3_-SCR process over V_2_O_5_/TiO_2_ catalysts discovered that the amount of gaseous SO_3_ products increased with temperature in this range. The presence of O_2_ in flue gas promoted the oxidation of SO_2_ and the formation of gaseous SO_3_ on the V_2_O_5_/TiO_2_ catalysts. On the catalyst surface, the major pathway for SO_2_ oxidation comprises oxidizing SO_2_ to generate adsorbed SO_3_, which either desorbs to form gaseous SO_3_ or interacts with NH_3_ to form sulfate. Sulfite is created when a tiny amount of SO_2_ interacts with NH_3_; this product was then quickly oxidized to (NH_4_)_2_SO_4_. At 180 °C, the amount of deposition products peaked, while below 230 °C, more NH_4_^+^ emerged. Additives that also strengthened the water and sulfur resistance of the rare-earth-based catalysts helped them conduct denitrification even better. Many researchers have prepared binary-loaded metal oxide catalysts using transition metals and doping with rare earth elements such as CoO,^9^ Cr_2_O_3_,^10^ and Sm_2_O_3_.^11^ This method boosted the denitrification activity of the original Ce-based catalysts as well as their resistance to water and sulfur.

In this paper, we prepared a Ce–La–V/TiO_2_ catalyst *via* a previously reported ball milling method.^12^ The effect of the V introduction on the denitrification performance of the catalyst as well as on the water and sulfur resistance was tested. It was found that the NO conversion still maintained 85% after 52 h of water and sulfur feeding. After stopping the water and sulfur, the NO conversion recovered to 99%. In addition, the mechanism of the resistance to SO_2_ toxicity at high temperatures was elucidated using various physicochemical characterization techniques and reaction kinetics analysis.

## 2 Experimental

### 2.1 Catalysts preparation

According to the chemical formula of Ce_10_La_2_V_*x*_Ti_88−*x*_ (where *x* is the mass ratio of V_2_O_5_ per 100 parts of catalyst), cerium carbonate, lanthanum carbonate, ammonium metavanadate and titanium dioxide were added and put in the nylon ball milling jar, appropriate amount of deionized water and zirconia balls were added. The volume of the ball milling jar was 530 mL, the inner diameter was 94.5 mm, the height was 93.5 mm, the weight of zirconia balls was 95 g, and the volume of water was 100 mL. Then the slurry was ball milled in KQM-Z/B planetary ball mill, the rotational speed and time were set (usually 500 rpm, 1 h), and the direction of rotation was changed once per 20 min, separation of the slurry from the zirconia balls at the end of the process, and the slurry was dried in an oven at 105 °C for 12 h. After drying, the slurry was calcined at 500 °C for 4 h to obtain the desired catalysts, and the corresponding catalysts were notated as 10Ce–2La–*x*V/Ti (*x* = 0.1, 0.3, 0.6, 0.9, 1.4 and 2.3). In addition, the catalyst was poisoned by passing 300 ppm SO_2_ and 5% H_2_O on top of a mixture of 500 ppm NO, 500 ppm NH_3_, 5% H_2_O and N_2_, which was allowed to stabilize and then reacted continuously for 4 h to obtain a poisoned sample, which was labeled as 10Ce–2La–*x*V/Ti–S.

### 2.2 Catalysts activity test

First, the prepared catalysts were ball-milled and attached to porous cordierite cylinders (commercial grade, *φ* 20 × *L* 50). After drying in an oven, the catalyst-loaded cordierite cylinders were placed in a quartz tube furnace to test the catalytic activity of the catalyst. The catalyst was loaded with a weight of 1 g. The gas mixture consisted of 500 ppm NO, 500 ppm NH_3_, 300 ppm SO_2_, 5% H_2_O, and 3% O_2_, with N_2_ as the equilibrium gas. The total gas flow rate was 2500 mL min^−1^ and the corresponding GHSV was 150 000 mL g^−1^ h^−1^. The flow of all the gases was controlled by a mass flowmeter. NO and NO_2_ concentrations at the outlet were monitored in real time using an ECOM flue gas analyzer (Germany). NO conversion, N_2_ selectivity and SO_2_ conversion were calculated from eqn (1)–(3):1
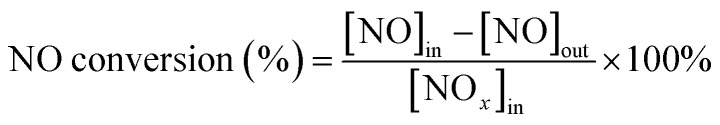
2
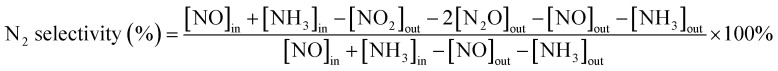
3
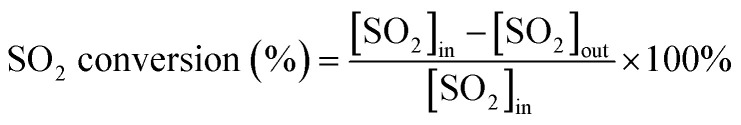
where the subscript ‘in’ and ‘out’ represent the inlet and outlet concentrations of each gas at steady state, respectively.

In order to visualize the differences in the catalytic activity of the samples more intuitively, the tests were conducted at a higher GHSV (150 000 mL g^−1^ h^−1^), keeping the NO conversion rate lower than 30%, and the surface activation energies of the different catalysts were calculated. Assuming that the NH_3_-SCR catalytic reaction is not controlled by diffusion, the SCR reaction rate normalized by the specific surface area of the catalyst can be calculated according to eqn (4)^13,14^4
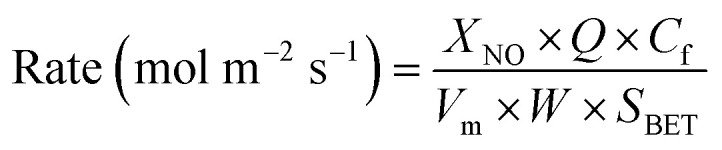
where *X*_NO_ is the NO conversion (%) at different temperatures, *Q* is the volumetric flow (2500 mL min^−1^), *V*_m_ is the molar volume of gas under standard conditions (22.4 L mol^−1^), *C*_f_ is the fed NO concentration (500 ppm), *W* is the weight of the catalyst (g), and *S*_BET_ is the specific surface area of the catalyst (m^2^ g^−1^).

Turn over frequency (TOF) can be calculated to compare the catalytic rate of different catalysts. Keeping the NO conversion below 30% (to ensure that all catalytically active sites are operational), the TOF value of NO on the active center Ce is calculated from eqn (5):^15,16^5
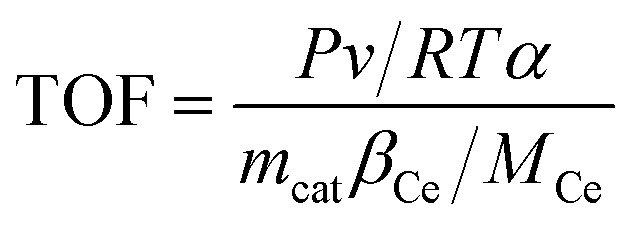
where *P* is the standard atmospheric pressure (1.01 × 10^5^ Pa), *v* is the flow of NO, *R* is the molar gas constant (8.314 J mol^−1^ K^−1^), *T* is the test temperature (K), *α* is the NO conversion (%), *m*_cat_ is the weight of the catalyst (g), *β*_Ce_ is the loading ratio of Ce (%) calculated using XPS data, and *M*_Ce_ is the molecular weight of cerium (140.1 g mol^−1^).

### 2.3 Characterization of catalysts

The physical composition of the samples was examined using a Thermo ARL SCINTAG X'TRA X-ray diffraction analyzer. Cu K-rays (wavelength 0.154056 nm) were used as its X-ray source, with a tube voltage of 40 kV and a tube current of 40 mA. Sample surface area, pore volume and pore size were measured by N_2_ adsorption and desorption using a Biaode SSA-7300 automatic pore size and surface area analyzer. X-ray photoelectron spectroscopy (XPS) was carried out using a Thermo Scientific K-Alpha electron spectrometer equipped with an Al Kα (*hv* = 1486.6 eV) radiation at an operating voltage of 12 kV and a reference pressure of 3 × 10^−7^ mbar. Temperature programmed desorption/reduction (TPD/TPR) experiments were carried out on a chemisorption apparatus (Micromeritics, AutoChem II 2920). TG and DSC quantities were measured using a simultaneous thermal analyzer (PerkinElmer STA-8000). N_2_ was used as the carrier gas, and the samples were heated up to 100 °C at a heating rate of 10 °C min^−1^ to pretreat moisture and other impurities on their surface, and then heated up to 800 °C at a heating rate of 10 °C min^−1^. The *in situ* IR tests were performed on an *in situ* FTIR spectrometer (Tensor 27). The tests were consisted by four parts: NH_3_ adsorption, NO + O_2_ adsorption, NO + O_2_ reaction with pre-adsorbed NH_3_ and NH_3_ reaction with pre-adsorbed NO + O_2_.

## 3 Results and discussion

### 3.1 Characterization of catalysts physical properties

#### 3.1.1 XRD

**Fig. 1:**
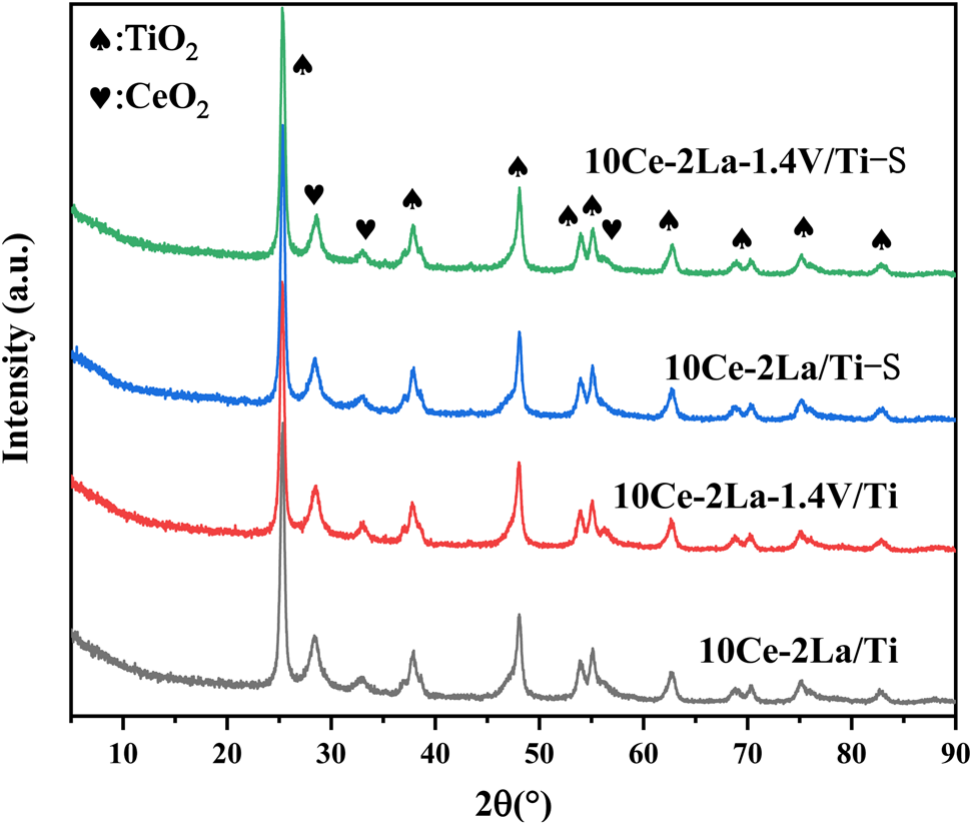
XRD spectra of 10Ce–2La–*x*V/Ti series catalysts before and after poisoning.

The physical phases present in the catalysts were analyzed by X-ray diffraction. As shown in Fig. 1, all samples contained peaks near 25.2°, 37.7°, 48.0°, 53.9°, and 54.9°, which belonged to anatase TiO_2_ (JCPDS no. 21-1272). In addition, weak diffraction peaks of CeO_2_ (JCPDS no. 43-1002) were detected near 28.6°, 33.0°, which indicated that some CeO_2_ was present on the catalyst surface. La_2_O_3_ and V_2_O_5_ diffraction peaks were not observed for all samples, indicating that they are poorly crystallized and highly dispersed on the surface of the catalysts, which allows the active components to interact more fully and thus enhance the catalytic activity.^17^ The absence of neither crystallographic changes nor the appearance of new diffraction peaks in the poisoned samples compared with the fresh samples may be due to the low content of sulfate species generated on the catalyst surface or that they were amorphous.

**Fig. 2:**
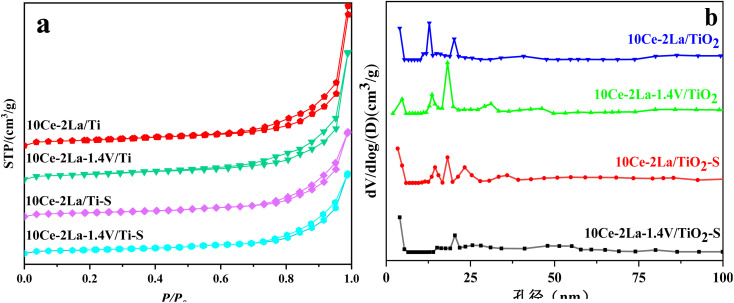
(a) N_2_ adsorption–desorption isotherms and (b) BJH pore size distributions of 10Ce–2La–*x*V/Ti series catalysts before and after poisoning.

Fig. 2 shows the N_2_ adsorption–desorption isotherms and BJH pore size distribution. As shown in Fig. 2(a), all samples showed typical H3 hysteresis loops at higher relative pressures in the latter half (*P*/*P*_0_ = 0.7–1.0), which is usually associated with capillary coalescence in the catalyst.^18^ The curves were a type IV isotherm according to the IUPAC classification, indicating that the catalysts had mesoporous structures. From Fig. 2(b), it is clear that all samples showed a wide range of pore sizes, further confirming that the catalysts are mesoporous and are made up of a variety of irregularly shaped particles stacked together. In addition, the specific surface area, total pore volume, and average pore size obtained from N_2_ adsorption–desorption isotherms were calculated (Table 1). These values were slightly reduced after adding V because it favored sintering. After poisoning, the catalyst's surface area, average pore volume, and total pore volume were drastically decreased, demonstrating that sulfate species had coated the catalyst's surface.^19^

**Table 1:** Structural information of 10Ce–2La–*x*V/Ti series catalysts

Samples	Surface area (m^2^ g^−1^)	Pore volume (cm^3^ g^−1^)	Diameter of hole (nm)
10Ce–2La/Ti	74.13	0.062	21.96
10Ce–2La–1.4V/Ti	71.81	0.056	16.55
10Ce–2La/Ti–S	57.51	0.037	15.73
10Ce–2La–1.4V/Ti–S	54.30	0.035	15.65

#### 3.1.2 TEM

**Fig. 3:**
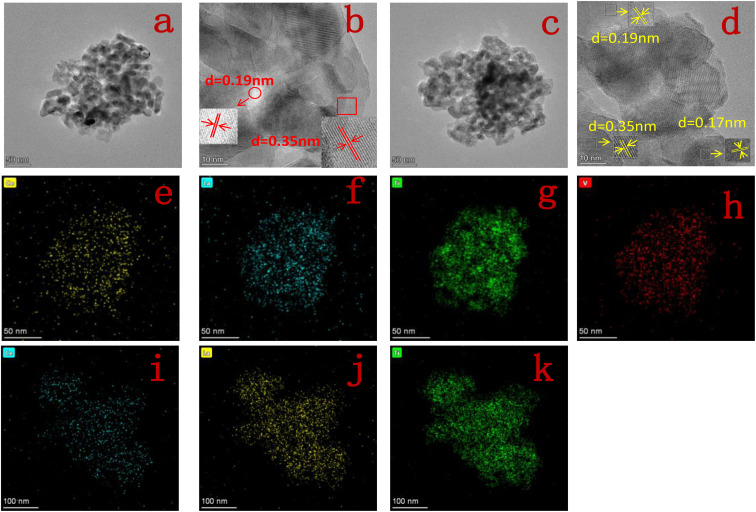
(a and b) TEM and HR-TEM image of 10Ce–2La/Ti; (c and d) TEM and HR-TEM image of 10Ce–2La–1.4V/Ti; (e–h) elemental distribution of Ce, La, Ti, V of 10Ce–2La–1.4V/Ti; (i–k) elemental distribution of Ce, La, and Ti of 10Ce–2La/Ti.

Fig. 3 shows the TEM images, HR-TEM images and elemental distributions of Ce, La, Ti and V for 10Ce–2La/Ti and 10Ce–2La–1.4V/Ti. From Fig. 3(a) and (c), it can be seen that the catalysts are porous structures formed by the stacking of nanoparticles with uneven sizes, which can exhibit good catalytic activity. In the HR-TEM images in Fig. 3(b) and (d), the crystal spacing of 0.35 nm corresponds to the (101) facets of anatase TiO_2_, 0.19 nm corresponds to the (220) crystalline facets of CeO_2_, and 0.17 nm can be indexed to the (202) crystalline facets of TiO_2_. Lattice fringes of lanthanum oxide and vanadium oxides were not found in any of the catalysts, confirming that they existed in an amorphous structure. It can also be observed that the elemental distribution of Fig. 3(e–h) and (i–k) shows that the elements Ce, La, Ti, and V are all uniformly distributed in the catalyst.^20^

### 3.2 Catalyst surface acidity

**Fig. 4:**
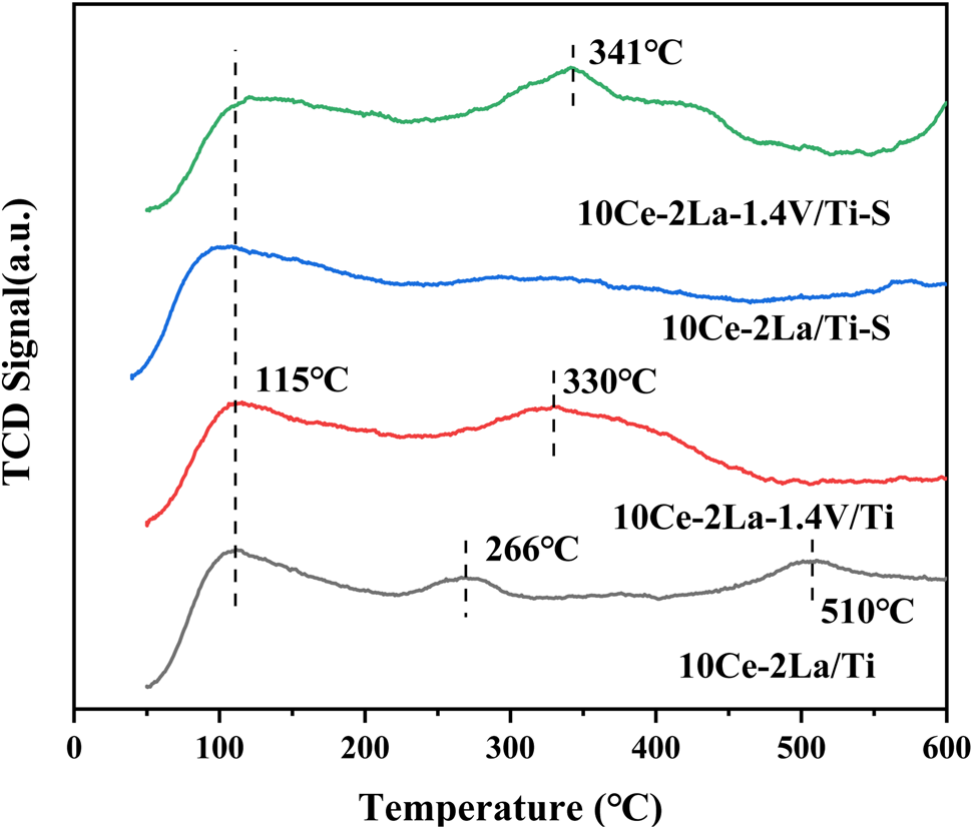
NH_3_-TPD spectra of 10Ce–2La–*x*V/Ti series catalysts before and after poisoning.

NH_3_-TPD was used to investigate the acidic properties of the catalyst surface (Fig. 4). While the peaks of medium and strong acidic sites in the temperature range of 200–500 °C were associated with the desorption of NH_4_^+^ from Brønsted acidic sites, the peaks of weak acidic sites below 200 °C corresponded to physisorbed NH_3_ on the catalyst. The peaks of strong acidic sites >500 °C were attributed to the desorption of liganded NH_3_ on Lewis acidic sites.^21^ By comparison, we found that the weak acidic site peaks did not change much after the introduction of V. However, the peak intensities and peak areas of the strong acidic sites in the catalysts increased significantly and the detachment peaks moved toward higher temperatures, which may be due to the interaction of the different active components, which in turn led to an increase in the length of the metal–oxygen coordination bonds and the surface acidity.^22^ Meanwhile the decrease in the peak area of the strong acidic sites indicates that the medium-strong and weak acidic sites play a greater role in the catalytic activity of the catalyst during the catalytic reaction. In addition, it was observed that the peak intensity of the weak acidic sites of the 10Ce–2La/Ti catalyst became smaller after poisoning, which may be due to the reaction of SO_2_ on the surface of the catalyst to generate sulfate to reduce the number of acidic sites, which led to the weakening of the catalyst's ability to adsorb NH_3_. In contrast, the peak position of catalyst 10Ce–2La–1.4V/Ti–S not only shifted toward high temperature, but also the peak intensity and peak area of the medium-strong acidic sites increased instead, indicating that the sulfation of the catalyst increased the NH_3_ uptake by the medium, which is one of the reasons that the catalyst exhibits good water and sulfur.^23^

### 3.3 Catalyst redox properties

**Fig. 5:**
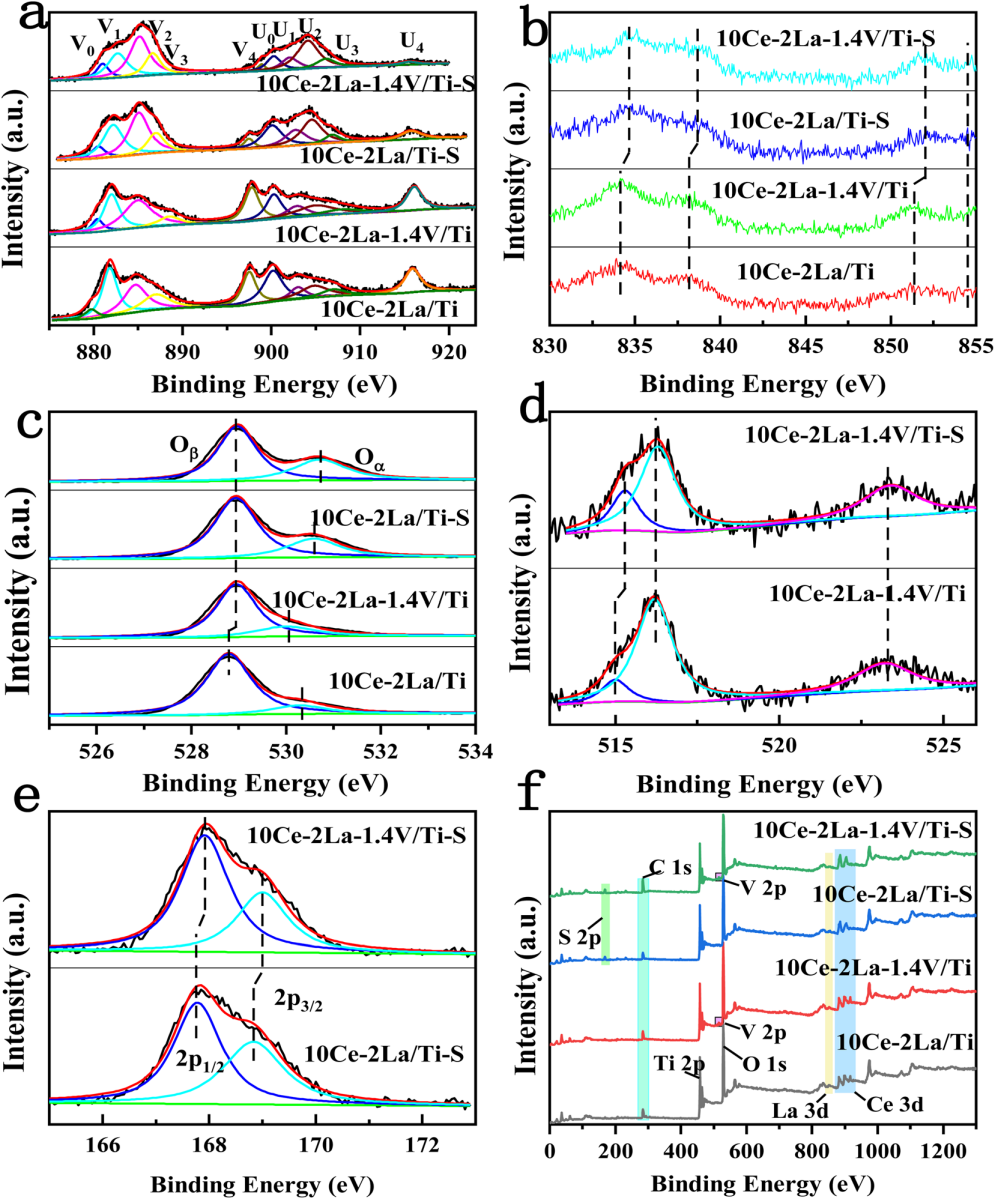
XPS spectra of 10Ce–2La/Ti and 10Ce–2La–1.4V/Ti catalysts and before and after poisoning. (a) Ce 3d; (b) La 3d; (c) O 1s; (d) V 2p; (e) S 2p; (f) full spectrum.

XPS was used to investigate the chemical environment of the catalyst surface elements. The XPS spectra of catalyst Ce 3d are shown in Fig. 5(a), in which ten peaks were fitted to the spectra of all the samples. These were identified with “U” and “V” for the 3d_5/2_ and 3d_3/2_ spin–orbit components of Ce, respectively, which were located at 879 eV (V_0_), 885 eV (V_2_), 900 eV (U_0_), and 905 eV (U_2_). These belong to the 3d^10^4f^1^ initial electronic state of Ce^3+^, and the remaining peaks were related to the 3d^10^4f^0^ state of Ce^4+^. The existence of the double peaks of V_0_/U_0_ and V_2_/U_2_ suggests the presence of partially-reduced oxygen vacancies in the catalyst.^24^ The fitted integrated peak area ratio of Ce^3+^ and total Ce was used to determine the relative content of Ce^3+^ on the catalyst surface, as shown in Table 2 by the formula Ce^3+^ (%) = (*S*_V_0__ + *S*_V_2__ + *S*_U_0__ + *S*_U_2__)/(*S*_U_ + *S*_V_) × 100%.^25^ After introducing V, Ce and La concentrations on the catalyst surface considerably rose, proving that V interacted with the active ingredients and had an impact on how many atoms were present there. The higher proportion of Ce^3+^, was attributed to an increase in the Ce concentration and also to the movement of free carriers on the catalyst surface towards the CeO_2_ surface, which had a higher binding energy (eV).^26^ A large fraction of Ce^3+^ on the catalyst surface enhanced the formation of oxygen vacancies, unsaturated chemical bonds, and charge imbalance, all of which boost the NO oxidation reaction.^27,28^Fig. 5(b) depicts the catalyst's La 3d XPS spectra, in which La 3d_5/2_ and La 3d_3/2_ peaks emerged in all spectra at 834 eV and 37 eV, as well as at 852 eV and 855 eV, respectively.^29^

**Table 2:** Surface element concentrations and Ce^3+^, O_α_, and V^4+^ ratios of catalysts

Samples	Surface atomic concentration (at%)						Ce^3+^/Ce	O_α_/O	V^4+^/V
	O	Ce	La	V	S	Ti			
10Ce–2La/Ti	71.94	2.74	0.28	—	—	25.04	42.14%	14.34%	—
10Ce–2La–1.4V/Ti	70.58	3.42	0.40	1.21	—	24.92	45.60%	21.40%	9.98%
10Ce–2La/Ti–S	70.53	2.39	0.25	—	3.75	23.08	53.49%	27.51%	—
10Ce–2La–1.4V/Ti–S	69.84	2.92	0.40	0.72	4.63	21.50	57.30%	34.07%	18.83%

Fig. 5(c) shows the O 1s XPS spectrum of the catalyst, in which peaks belonging to lattice oxygen and surface unsaturated oxygen (labeled O_β_ and O_α_), can be observed at positions 528.7–529 eV and 530.1–530.7 eV. Because surface unsaturated oxygen O_α_ has a higher migration rate than lattice oxygen O_β_, O_α_ increased the number of surface oxygen vacancies, which were more favorable to the SCR reaction.^30^ The relative atomic percentages of these oxygen species on the catalyst surface were estimated from the areas of the fitted peaks, as listed in Table 2. The O_α_/(O_α_ + O_β_) ratio on the catalyst surface significantly increased after adding V, which is consistent with the Ce 3d XPS analysis and is one of the reasons for the good activity of the catalyst. Fig. 5(d) showed the V 2p XPS spectra before and after catalyst poisoning, in which the V^4+^ peak was located at 515.2 eV, and the V^5+^ peaks were located at 516.4 eV and 523.1 eV. This indicates that V on the catalyst surface mainly existed as V^5+^.^31^ The ratio of V^4+^/(V^4+^ + V^5+^) on the catalyst surface increased significantly after poisoning. Ce^3+^ also increased significantly, but the concentrations of Ce and V, the main active components on the surface of the catalyst, decreased. This was because SO_2_ reacted with the active components to produce sulfate in the presence of O_2_.^7^ Similarly, the significant increase in the O_α_/(O_α_ + O_β_) ratio on the surface after catalyst poisoning was because the higher Ce^3+^ ratio promoted the creation of more surface oxygen vacancies. This was because oxygen from sulfate species was formed on the sample surface after sulfation, as well as chemisorbed oxygen.^32^Fig. 5(e) shows the S 2p XPS spectra of the catalyst before and after poisoning, in which peaks belonging to the spin–orbit components of S^6+^ 2p_3/2_ and S^6+^ 2p_1/2_ can be observed at 167.7–168 eV and 168.8–169 eV after fitting. This implies that S in +6 valence state was mostly present on the catalyst surface as SO_4_^2−^.^33,34^ After poisoning, the XPS peaks of all elements on the catalyst changed to higher binding energies, showing that the electron cloud density around each element was lowered to varying degrees. All were involved in the sulfation reaction. Peaks of Ce, La, O, Ti, V, S and C were observed in the full spectrum of the catalyst in Fig. 5(f).

**Fig. 6:**
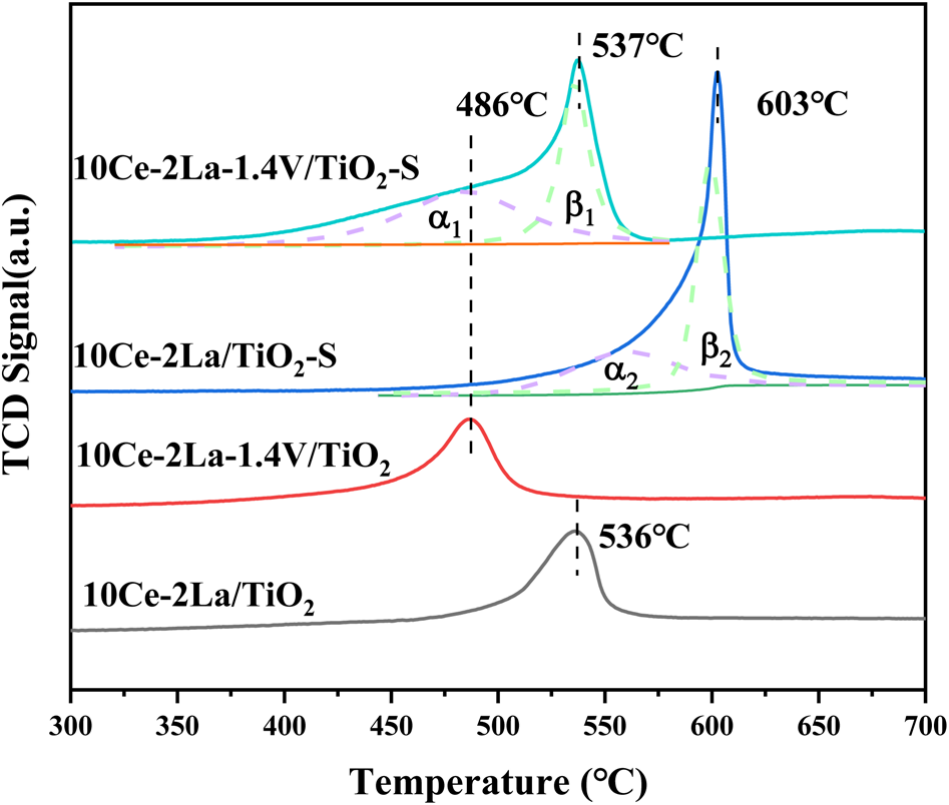
H_2_-TPR spectra of 10Ce–2La–*x*V/Ti series catalysts.

As shown in Fig. 6, the redox properties of catalysts before and after 10Ce–2La/Ti and 10Ce–2La–1.4V/Ti poisoning were tested using H_2_-TPR. Measurements were performed using CuO as the standard, and the hydrogen consumption for the reduction peaks between 400 °C and 700 °C was quantitatively calculated (Table 3). 10Ce–2La/Ti displayed a large H_2_ reduction peak, which was attributed to the reduction of surface-liganded unsaturated Ce^4+^, which played an important role in the oxidation reaction.^35^ After introducing V, the catalyst reduction peaks shifted toward lower temperatures, and the H_2_ consumption increased significantly (1.37 → 1.51 mmol g^−1^). This indicates strong interactions between V oxides and Ce oxides on the catalyst, which enhanced the reducing properties of the catalyst, which promoted catalytic cycling during SCR reaction.^36^ A strong peak was observed at 603 °C in the poisoned 10Ce–2La/Ti–S sample, which was shifted to a higher temperature relative to the fresh sample. This shift was primarily due to the reduction of S^6+^ to S^4+^ in sulfate, along with the reduction of certain metal oxides.^37^ To study the degree of metal oxide reduction in the catalyst 10Ce–2La–1.4V/Ti–S, the peaks were divided into α- and β-peaks by fitting, and the fitted area ratio was used to calculate the amount of hydrogen consumed for each peak. The α peak was associated with the reduction of metal oxides on the catalyst surface, while the β peak was related to the reduction process of sulfate species.^36^ The position of the metal oxide reduction peak in 10Ce–2La–1.4V/Ti–S was essentially unchanged, and the hydrogen consumption of *α*_1_ was larger than that of *α*_2_ (1.54 > 0.89 mmol g^−1^). That of *β*_1_ was less than that of *β*_2_ (1.00 < 1.157 mmol g^−1^), indicating that co-doping V with 10Ce–2La/Ti limited the interactions between SO_2_ and active metal oxide.

**Table 3:** Catalyst H_2_-TPR peak positions and corresponding H_2_ consumption

Samples	Peak position (°C)	H_2_ consumption (mmol g^−1^)
10Ce–2La/Ti	536	1.37
10Ce–2La–1.4V/Ti	486	1.51
10Ce–2La/Ti–S	603	2.05
10Ce–2La–1.4V/Ti–S	537	2.54

### 3.4 SO_2_ desorption and reaction

**Fig. 7:**
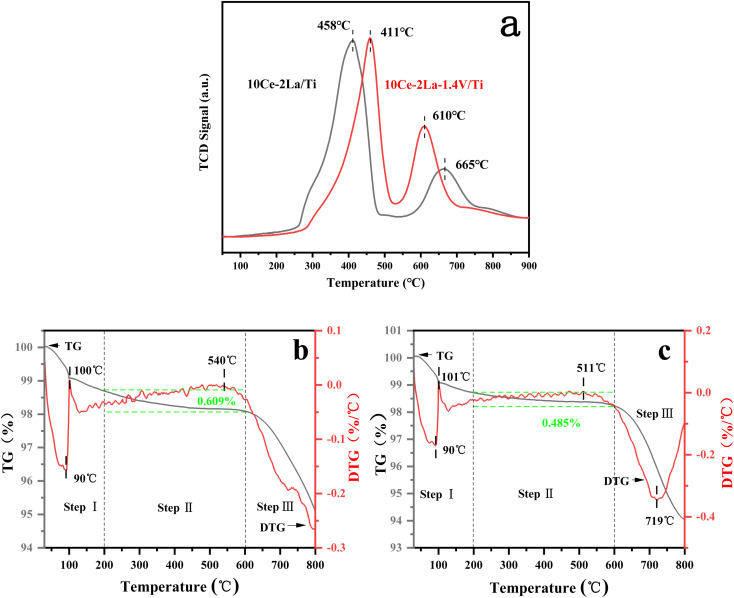
SO_2_-TPD curves of fresh catalysts and TG/DTG curves of poisoned catalysts. (a) SO_2_-TPD curves; (b) 10Ce–2La/Ti; (c) 10Ce–2La–1.4V/Ti.

SO_2_-TPD was used to investigate the adsorption capacity of the catalyst toward SO_2_. As shown in Fig. 7(a), the catalyst contained two main desorption peaks in the temperature ranges of 400–460 °C and 600–670 °C. The low-temperature peaks were related to the desorption of SO_2_ from the catalyst surface,^38^ while the high-temperature peaks were related to the desorption of SO_2_ generated by the decomposition of sulfate species. The peak of 10Ce–2La/Ti at 665 °C represented the decomposition of cerium sulfate.^39^ In comparison, the desorption peak of 10Ce–2La/Ti at low temperature has a larger peak area, implying that more SO_2_ molecules are absorbed on the surface than 10Ce–2La–1.4V/Ti, the addition of V reduced the amount of SO_2_ molecules absorbed on the surface of the catalyst in order to alleviate competition between SO_2_ and NO adsorption, and to prevent the occupation of more active sites. The addition of V enhanced the strength of the catalyst's high-temperature peak, indicating that SO_2_ molecules were adsorbed onto V_2_O_5_ species, shielding the catalyst's major active sites.^40^

To study the substances generated on the surface of the samples during the catalytic reaction, the poisoned samples were analyzed using thermogravimetric analysis, and the results are shown in Fig. 7(b) and (c). The two samples' weight reduction procedure was separated into three steps: step I (40–200 °C) was mostly ascribed to the desorption of water from the samples; step II (200–600 °C) was attributed to the breakdown of ammonium sulfate and ammonia bisulfate on the catalyst surface; and step III (600–800 °C) to the decomposition of sulfates in the samples.^36,41^ 10Ce–2La–1.4V/Ti–S showed an absorption peak related to the decomposition of sulfate (511 °C) and an exothermic peak related to the phase transition of the composite metal oxide (719 °C) above 500 °C. The 10Ce–2La/Ti–S catalyst had a higher decomposition temperature (540 °C), which was attributed to the low decomposition temperature of VOSO_4_ generated by SO_2_ with V. The 10Ce–2La/1.4V/Ti–S catalyst had the highest decomposition temperature (540 °C). In addition, the weight loss of ammonium sulfate or ammonia bisulfate on the surface of the 10Ce–2La–1.4V/Ti–S catalyst was larger (0.609%) than that of 10Ce–2La/Ti–S (0.485%). It is shown that the introduction of V protects the main active component of the catalyst from reacting with SO_2_ and inhibits the formation of sulfate species on the catalyst surface, thus enhancing the water and sulfur resistance of the catalyst.

### 3.5 *In situ* infrared spectroscopy of catalysts

**Fig. 8:**
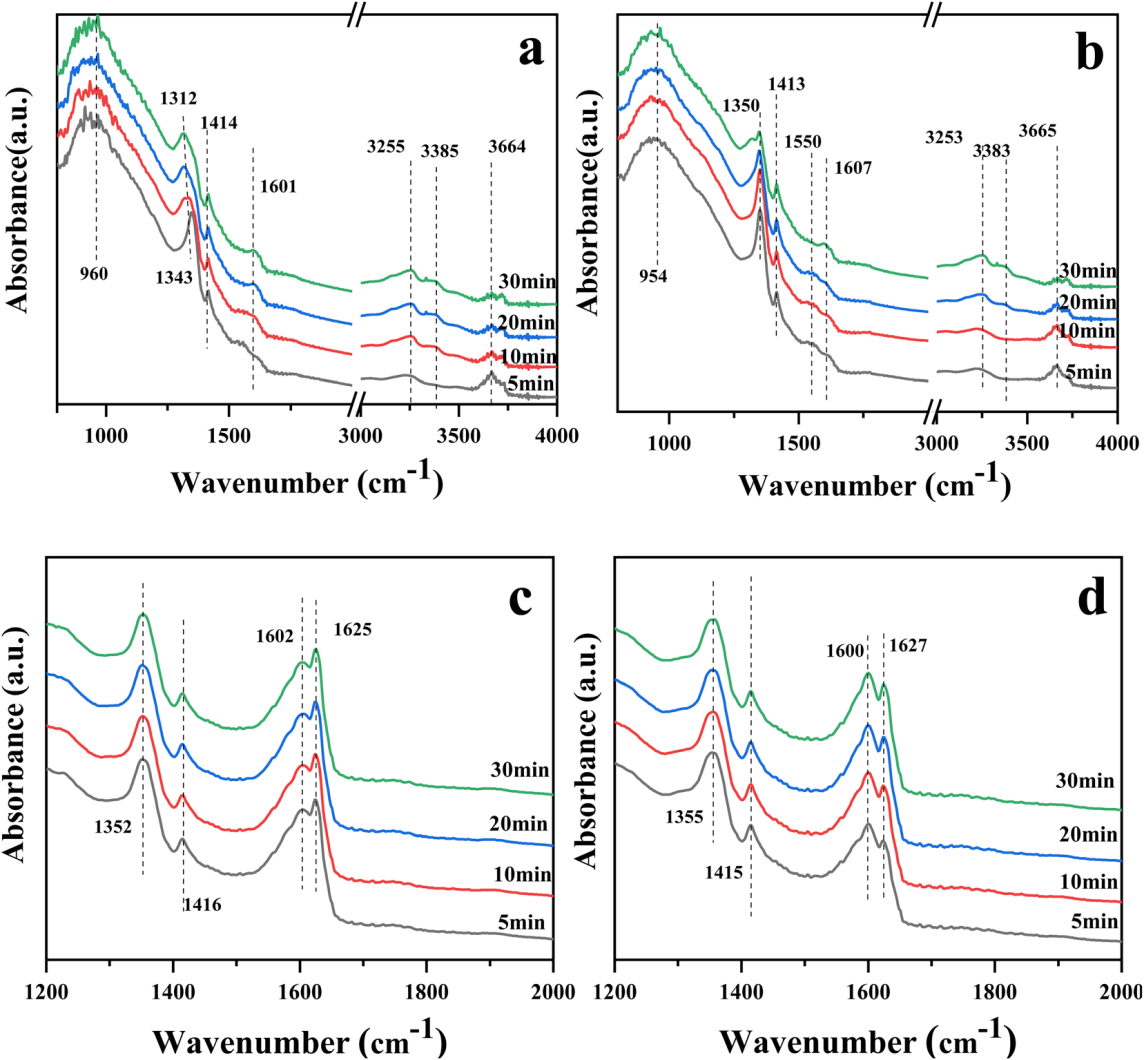
*In situ* DRIFTS spectra of NH_3_ or NO + O_2_ adsorption on catalysts. (a) NH_3_ adsorption on 10Ce–2La/Ti, (b) NH_3_ adsorption on 10Ce–2La–1.4V/Ti, (c) NO + O_2_ adsorption on 10Ce–2La/Ti, and (d) NO + O_2_ adsorption on 10Ce–2La–1.4V/Ti.

Fig. 8(a) and (b) show the *in situ* DRIFTS spectra of catalysts 10Ce–2La/Ti and 10Ce–2La–1.4V/Ti after adsorbing NH_3_ (500 ppm) for 30 min. The peaks near 954 (960) cm^−1^ for both samples were related to weakly-adsorbed gas-phase NH_3_.^42,43^ Absorption peaks near 1312–1343 (1350) cm^−1^ and 1414 (1413) cm^−1^ belonged to NH_4_^+^ at Brønsted acid sites.^44,45^ The asymmetric bending vibration of NH_3_ coupled to Lewis acid sites was responsible for the lower absorption peak at 1601 (1607) cm^−1^.^44^ The peak at 3253–3385 cm^−1^ was attributed to the stretching vibration of NH_3_ at the Lewis acid sites.^46^ 10Ce–2La–1.4V/Ti shows a weak adsorption band near 1550 cm^−1^, which is due to the oxidation of NH_3_ on the catalyst surface by surface-active oxygen to generate an important intermediate product, –NH_2_, in the catalytic reaction –NH_2_ facilitates the conversion of NO, and promotes the smooth progress of the SCR reaction.^47^ In comparison, the intensity of the peaks related to the Brønsted acidic sites increased after the introduction of V, and NH_3_ was more easily activated. This indicates that Brønsted acids dominated the reaction, and the addition of V increased the proportion of Brønsted acids.

The DRIFTS spectra of the catalysts 10Ce–2La/Ti and 10Ce–2La–1.4V/Ti after 30 minutes of NO (500 ppm) + O_2_ adsorption are shown in Fig. 8(c) and (d). Nitrate bridged ions and free nitrate ions, respectively, were responsible for the bands at 1355 (1352) cm^−1^ and 1600 (1602) cm^−1^.^48^ The absorption peak belonging to the nitrite species *trans*-N_2_O_2_^2−^ was located at 1415 cm^−1^. Weakly adsorbed gas-phase NO_2_ was thought to be active in the band at 1625–1627 cm^−1^ because it may react quickly with gaseous NH_3_.^45^

**Fig. 9:**
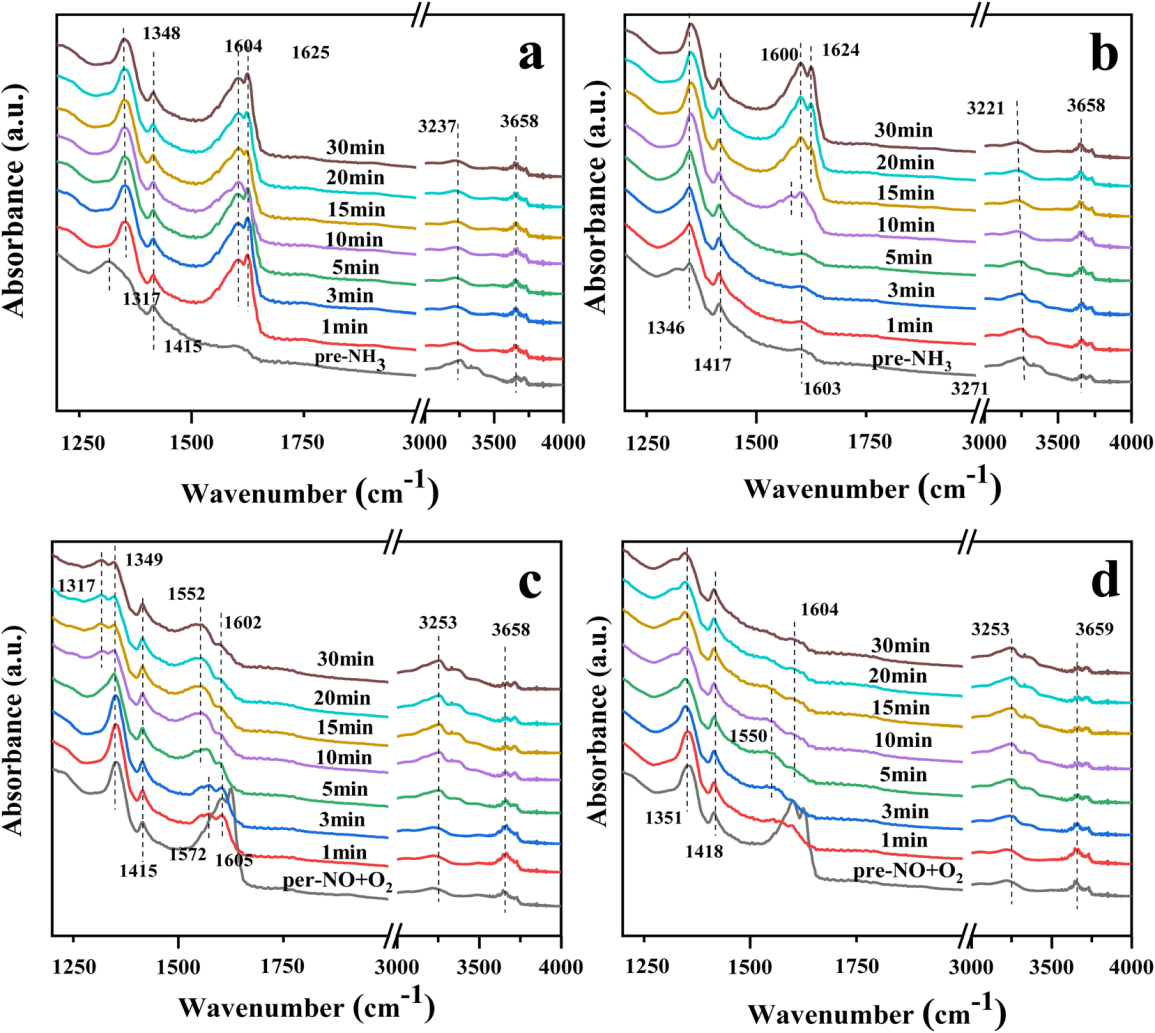
*In situ* DRIFTS spectra of NH_3_(NO + O_2_) reaction with pre-adsorbed NO + O_2_(NH_3_) on the catalyst. (a) Reaction of NO + O_2_ on 10Ce–2La/Ti with pre-adsorbed NH_3_; (b) reaction of NO + O_2_ on 10Ce–2La–1.4V/Ti with pre-adsorbed NH_3_; (c) reaction of NH_3_ on 10Ce–2La/Ti with pre-adsorbed NO + O_2_; (d) reaction of NH_3_ on 10Ce–2La–1.4V/Ti with pre-adsorbed NO + O_2_ reaction.

Fig. 9(a) and (b) show the DRIFTS profiles of the catalysts 10Ce–2La/Ti and 10Ce–2La–1.4V/Ti after 30 min of pre-adsorption of NH_3_ (500 ppm) followed by NO (500 ppm) + O_2_ (3%) adsorption for 30 min. In the NH_3_ adsorption curve, the peaks near 1317 and 1415 (1417) cm^−1^ belonged to NH_4_^+^ on the Brønsted acid sites, and the intensity of the peak at 1415 (1417) cm^−1^ did not change greatly with the passage of NO + O_2_. This was attributed to the coincidence of the peak at 1600 (1604) cm^−1^ for the NH_3_ and Lewis acid sites with the stronger peaks of rapidly-generated nitrate at 1604 and 1625 cm^−1^ after flowing NO + O_2_. This indicates that pre-adsorbed NH_3_ did not directly react with gas-phase NO and that the NH_3_-SCR did not involve gas-phase NO. With the passage of NO + O_2_, the intensity of the 1415 (1417) cm^−1^ peak did not change significantly, because, at this time, the 1600 (1604) cm^−1^ peaks of NH_3_ and Lewis acid sites overlapped with those of the stronger nitrate peaks at 1604 and 1625 cm^−1^. This suggests that pre-adsorbed NH_3_ did not react directly with gas-phase NO and that the NH_3_-SCR reaction did not proceed *via* the Eley–Rideal mechanism.

Fig. 9(c) and (d) show the DRIFTS spectra of the catalysts 10Ce–2La/Ti and 10Ce–2La–1.4V/Ti pre-adsorbed with NO (500 ppm) + O_2_ (3%) for 30 min and then allowed to adsorb NH_3_ (500 ppm) for 30 min. Prior to the passage of NH_3_, peaks attributed to nitrates were observed at 1349 (1351) cm^−1^, 1624 (1625) cm^−1^, 1415 (1418) cm^−1^, and 1600 (1604) cm^−1^, similar to those in Fig. 8(a) and (b).^49^ The nitrate peaks at 1602 and 1625 cm^−1^ disappeared rapidly upon the passage of NH_3_, it indicates that adsorbed NO can react with NH_3_ in the adsorbed state, so the reaction mechanism is Langmuir–Hinshelwood mechanism. In an L-H reaction mechanism, the reaction orders of both NO and NH_3_ are zero.^50^

### 3.6 Catalyst denitrification performance testing

**Fig. 10:**
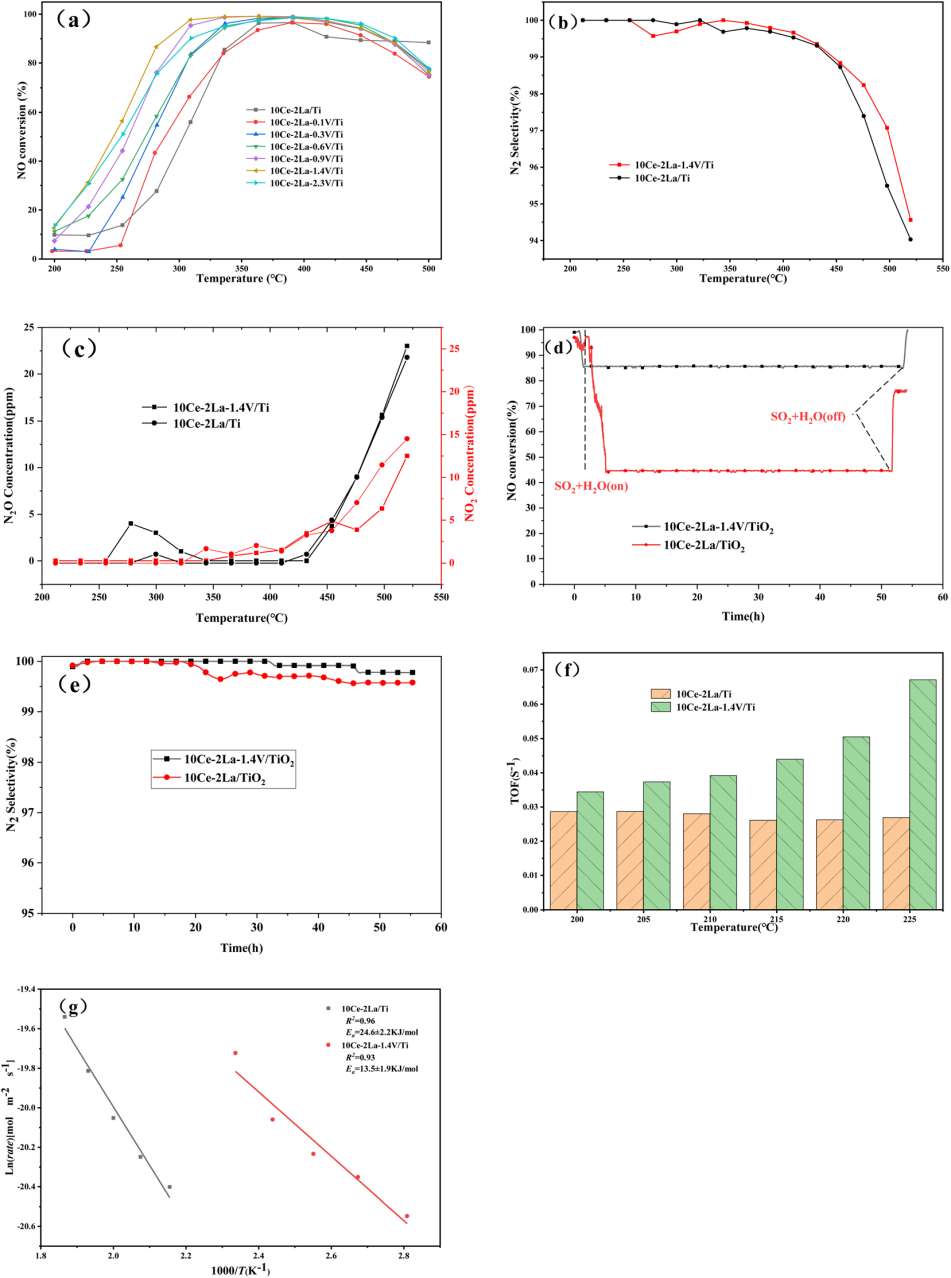
Catalytic performance *versus* temperature curves of 10Ce–2La–*x*V/Ti series catalysts. (a) NO removal efficiency *versus* temperature transformation curves at different weight ratios V; (b) N_2_ selectivity at different temperatures; (c) effect of temperature on the concentration of NO_2_ and N_2_O production; (d) stability test of the catalysts for water and sulfur resistance; (e) N_2_ selectivity at different time; (f) TOF values (200–225 °C). (g) Arrhenius plots.

Fig. 10(a) shows the NO conversion *versus* temperature curves of the catalysts after adding different ratios of V. The overall activity of the catalyst was improved after adding V, and the starting temperatures were all lower. As the weight percent of V increases, the catalyst denitrification efficiency improved and then became worse, and the temperature window gradually became wider. The optimal catalytic efficiency was obtained when the weight ratio of V_2_O_5_ was 1.4, and the denitrification efficiency remained above 80% in the temperature range of 270–490 °C. The NO conversion reached a maximum of 99% at 330 °C, and the catalytic activity was greatly improved compared with the original rare-earth-based catalyst. In addition to catalytic activity, N_2_ selectivity is another important index for evaluating a catalyst's performance. As the temperature of the reaction increased, N_2_O and NO_2_ were produced, causing the N_2_ selectivity to slowly decrease, as shown in Fig. 10(b) and (c). In the reaction temperature interval, the N_2_ selectivity and N_2_O concentration of catalyst was slightly improved by the addition of V. The lower N_2_O generation resulted in better overall N_2_ selectivity than the original catalyst.

The stability of the catalyst was tested by passing 300 ppm SO_2_ under constant test conditions and the results were shown in Fig. 10(d). After 2.5 h of the water and sulfur feeding, the NO conversion of 10Ce–2La/Ti decreased from the initial 97% to 44%, which maintained almost unchanged for 52 h. If the feeding of water and sulfur was cut off, the NO conversion recovered to 78%. Compared with 10Ce–2La/TiO_2_, the V-doped catalyst had a higher NO conversion in the presence of water and sulfur, which remained above 85% and recovered to 99% after shutting down water and sulfur. Fig. 10(e) shows the N_2_ selectivity comparison in the water and sulfur resistance stabilization experiments. Both the two catalysts showed a high N_2_ selectivity over than 99% and the N_2_ selectivity was not influenced by water and sulfur. It can also seen that the N_2_ selectivity was slightly improved by the addition of V. The turnover frequency (TOF) value calculated from eqn (5) is shown in Fig. 10(f). The 10Ce–2La–1.4V/Ti catalyst displayed better TOF values at each temperature, demonstrating that single Ce atoms on the catalytic surface were the primary active sites and displayed the best intrinsic activity under identical circumstances. Additionally, the reaction rate of NO on each square meter of a catalyst within the investigated temperature range was calculated according to eqn (4). It was plotted as ln(*rate*) against 1000/*T*, and the Arrhenius activation energy of the reaction was determined by fitting. As shown in Fig. 10(g), the surface activation energy of 10Ce–2La/Ti was 24.6 kJ mol^−1^, which was higher than that of 10Ce–2La–1.4V/Ti (13.5 kJ mol^−1^). The lower activation energy suggests an improved denitrification efficiency.

## 4. Conclusion

V-doped 10Ce–2La–*x*V/Ti catalysts were prepared by a ball milling method, and it was found that the rare earth Ce exerted a synergistic effect with V. The composite carrier-type oxides exhibited a good activity window and denitrification performance, as well as excellent water and sulfur resistance when the weight ratio of V_2_O_5_ was 1.4. The prepared catalysts showed mesoporous structures, and the ball milling method enhanced the interactions between different active components, resulting in good dispersion of the active components. V doping improved the surface acidity of the catalysts, and Brønsted acids on the surface of the catalysts played the dominant role. The greater number of acid sites adsorbed more NH_3_ that participated in the reaction and also prevented SO_2_ poisoning. The Ce^3+^ and O ratios on the catalyst surface were also enhanced by the addition of V, which was significant for the SCR-NH_3_ reaction. V limited the interactions between SO_2_ and the main active component (metal oxides), reduced the amount of SO_2_ adsorbed on the catalyst surface, and inhibited the formation of sulfate species on the catalyst surface. These all improved the sulfur resistance of the catalyst. The NH_3_-SCR on the catalyst surface proceeded *via* the Langmuir–Hinshelwood mechanism, with Brønsted acid sites playing the dominant role. The better denitrification activity and sulfur resistance of 10Ce–2La–*x*V/Ti catalysts make them promising for various applications.

## Author contributions

Yang Liu: writing – original draft, data curation, formal analysis, investigation, methodology. Na Wang: conceptualization, data curation, formal analysis, funding acquisition, investigation, methodology, project administration, supervision, validation, writing – review & editing. Huidong Xie: investigation, methodology, project administration, supervision. Yepeng Sun: data curation, formal analysis. Liang Zhang: investigation, methodology. Kaiyue Yang: investigation, methodology. Chang Yang: data curation, formal analysis. Chengmin Ge: conceptualization, funding acquisition, supervision, validation.

## Conflicts of interest

The authors declare that they have no known competing financial interests or personal relationships that could have appeared to influence the work reported in this paper.

## Supplementary Material
